# The Impact of COVID-19 Infection on a Neurologically Compromised Male With Fahr’s Disease Presenting With Acute Delirium and Aspiration Pneumonia: A Case Report

**DOI:** 10.7759/cureus.24233

**Published:** 2022-04-18

**Authors:** Rubal Bhangal, Jasmine K Sandhu, Zaryab Umar, Deesha Shah, Nso Nso

**Affiliations:** 1 Internal Medicine, Icahn School of Medicine at Mount Sinai / NYC Health + Hospitals Queens, New York, USA; 2 Internal Medicine, Icahn School of Medicine at Mount Sinai, Queens Hospital Center, New York, USA; 3 Medicine, Icahn School of Medicine at Mount Sinai / NYC Health + Hospitals Queens, New York, USA

**Keywords:** delirium, covid-19, toxic metabolic encephalopathy, sepsis, fahr’s disease or fahr’s syndrome

## Abstract

Fahr’s disease or idiopathic basal ganglia calcification is a rare, sporadic, genetically dominant, and inherited neurological condition that manifests with dysphagia and Parkinson’s disease. The computed tomography (CT) scan is the method of choice to diagnose basal ganglia calcifications seen in Fahr’s disease. This case report elaborates on the emergency management of a 58-year-old male patient with acute respiratory distress, acute delirium, schizophrenia, Fahr’s syndrome, and history of severe acute respiratory syndrome coronavirus 2 (SARS-CoV-2) (coronavirus disease 2019 or COVID-19) infection. The patient’s chest X-ray, laboratory workup, and vital signs were suggestive of aspiration pneumonia-induced sepsis and acute hypoxemic respiratory failure. Post-admission antibiotic management reduced sepsis complications without improving the altered mental status. A comprehensive clinical assessment suggested the attribution of Fahr’s disease to the patient’s aspiration pneumonia and other clinical complications. In addition, COVID-19 infection, sepsis-induced inflammatory processes, and pre-existing neurological compromise possibly deteriorated the patient’s neurological outcomes, overall prognosis, and recovery.

## Introduction

Fahr’s disease or syndrome is a rarely reported sporadic neurological condition with an autosomal dominant inheritance pattern that develops with the abnormal deposition of calcium phosphate and carbonate in the basal ganglia, cerebellar subcortical white matter, cerebral cortex, dentate nucleus, hippocampus, and thalamus [1]. The other predominant locations of idiopathic calcification include the lenticular nucleus, brain stem, cerebellum, subcortical white matter, centrum semiovale, thalamus, dentate nucleus, caudate, and putamen [1]. Fahr's syndrome was first recognized in 1930, and it has a prevalence of less than 1:1,000,000, with the majority of cases being described in males ranging from 30 to 60 years old [2-3]. Fahr’s syndrome leads to a progressive decline in mental health, early mortality in children, psychiatric symptoms in young adults, and dementia in middle-aged patients [4]. The commonly reported manifestations of this disease include extrapyramidal symptoms, orofacial dyskinesia, chorea, ataxia, oromandibular/bulbar dystonia, tremors, and parkinsonism [5]. The minor complications include gait disorder, psychiatric features, pyramidal signs, speech impairment, ataxia, cognitive decline, athetosis, eye impairment, seizures, headache, movement disorder, and decline in motor function [5]. Oropharyngeal dysphagia in Fahr’s disease progresses due to a rapid decline in the function of the stomatognathic system [6]. Recent studies reveal the role of the XPR1, PDGFB, PDGFRB, and SLC20A2 genes in triggering primary familial brain calcification in patients with Fahr’s disease [4,7]. In addition, Fahr’s syndrome potentially dysregulates the phosphate metabolism, blood-brain barrier (BBB), and cAMP pathway, which adds to the incidence of hypoparathyroidism and cerebral circulatory disturbances [1,3,8-10]. A computed tomography (CT) scan is a method of choice to diagnose calcifications in the dentate nuclei, corona radiata, thalami, and lentiform nuclei, while the non-calcified inflammatory processes are detected by magnetic resonance imaging (MRI) in the setting of Fahr’s disease [11]. In addition, the assessment of other associated conditions, including lipoid proteinosis, mitochondrial myopathy, tuberculous sclerosis, neuroferritinopathy, brucellosis infection, hyperparathyroidism, pseudo-hypothyroidism, secondary hypoparathyroidism, and idiopathic hypoparathyroidism is paramount to ruling out Fahr’s disease [12]. The treatment algorithms for Fahr’s disease aim to manage neuropsychiatric symptoms and their underlying etiologies [13]. This case study pertains to a 58-year-old male patient with a history of Fahr’s disease, acute delirium, aspiration pneumonia, and recent exposure to severe acute respiratory syndrome coronavirus (SARS-COV)-2 infection.

## Case presentation

**Figure 1:**
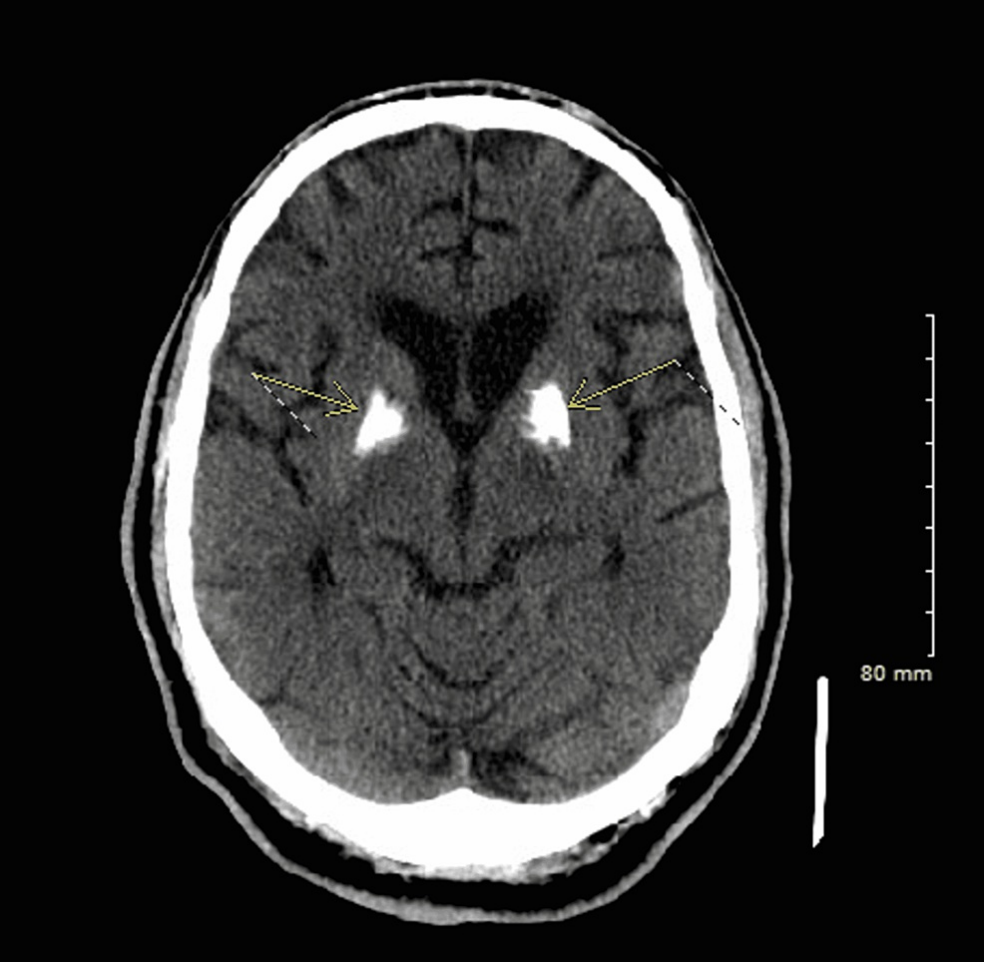
CT of the head shows mild, vague patchy areas of low attenuation in the periventricular white matter, likely ischemic/hypertensive in nature Dense bilateral near-symmetric areas of increased density/mineralization in the left and right basal ganglia and to a lesser degree, the left and right posterior thalamus dentate nuclei are seen as well, likely representing Fahr’s disease.

**Figure 2:**
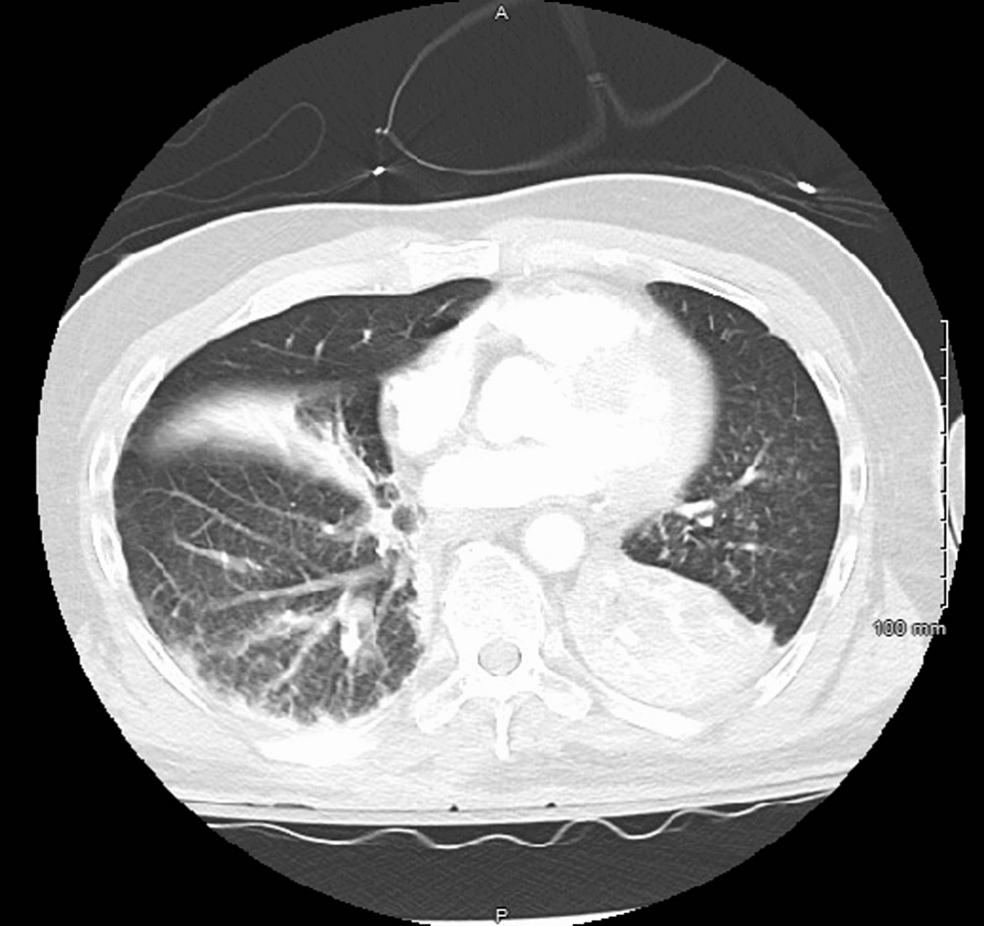
CT chest pulmonary angiogram shows atelectasis in the right middle lobe and bilateral lower lobes, significantly worse in the left lower lobe

We report the case of a 58-year-old African American male with a past medical history of hypertension, diabetes mellitus, atrial fibrillation, coronary artery disease, cerebrovascular accident, schizophrenia, and benign prostatic hypertension who presented to the emergency department (ED) from a nursing home with altered mental status and respiratory distress. While being transported in the ambulance, the patient was hypoxic with an oxygen saturation of 80%. His oxygen saturation improved to 93% when provided with 4 liters of oxygen supplementation via nasal cannula. He then had a cardiac arrest and cardiopulmonary resuscitation was initiated, however, return of spontaneous circulation was achieved without any medications. In the ED, he was tachycardic, tachypneic, hypotensive, and had a fever of 101.5 F. He was very lethargic and nonverbal but moving all four extremities. Lung examination was significant for bilateral diffuse rhonchi. Point-of-care ultrasound examination revealed left lower lobe consolidation. Initial labs revealed a leukocytosis of 22.81 x 103/mcL (normal range: 4.80-10.80 x 103/mcL), hyperkalemia of 5.8 mmol/L (normal range: 3.5-5.1 mmol/L), and mild acute kidney injury (AKI) with a creatinine of 1.28 mg/dL (0.7-1.2 mg/dL). Lactate was mildly elevated at 1.7 mmol/L (normal range: 0.6-1.4 mmol/L). See Table 1. SARS-CoV-2 (coronavirus disease 2019 (COVID-19)) PCR test was negative. CT of the head revealed mild, vague patchy areas of low attenuation in the periventricular white matter, likely ischemic/hypertensive in nature. In addition, CT of the head showed dense bilateral near-symmetric areas of increased density/mineralization in the left and right basal ganglia and to a lesser degree, the left and right posterior thalamus dentate nuclei as well, likely representing Fahr’s disease (Figure 1). CT chest pulmonary angiogram showed atelectasis in the right middle lobe and bilateral lower lobes, significantly worse in the left lower lobe (Figure 2). Blood, urine, and sputum cultures revealed no bacterial growth. Based on these findings, the patient met the criteria for sepsis, and antibiotics treatment was initiated.

**Table 1:** Initial laboratory findings BUN: blood urea nitrogen

Lab	Normal Reference Range with units	Value
White blood cells	4.80-10.80 x 10^3^/mcL	22.81
Hemoglobin	14.0-18.0 g/dL	9.8
Platelet	150-450 x 10^3^/mcL	522
Lactate	0.6-1.4 mmol/L	1.7
Sodium	136-145 mmol/L	137
Potassium	3.5-5.1 mmol/L	5.8
BUN	6-23 mg/dL	30
Creatinine	0.7-1.2 mg/dL	1.7
Albumin	3.5-5.2 g/dL	2.9

The patient was admitted for acute hypoxic respiratory failure and sepsis secondary to aspiration pneumonia. Initially, he required oxygen supplementation with a high-flow nasal cannula, however, his supplemental oxygen was de-escalated to the nasal cannula and eventually room air within two days. He received 10 days of piperacillin-tazobactam and vancomycin as well as three days of azithromycin. His leukocytosis and acute kidney injury (AKI) improved, and he remained afebrile for the remainder of his hospital stay. As aspiration pneumonia improved, the patient’s mental status remained unchanged. Electroencephalogram showed the posterior dominant rhythm consisting of 6-hertz theta waves with an amplitude of 25 uV. Diffuse slowing, but no focal slowing or epileptiform waves are seen. As per the patient’s nursing home, he had contracted the SARS-CoV-2 (COVID-19) infection one month prior to presentation, and since then he had not been behaving like himself and was more non-verbal than usual. Neurology was consulted and assessed that the patient’s altered mental status was likely due to acute delirium in the setting of sepsis and pre-existing Fahr’s disease. Psychiatry was also consulted and recommended minor dosage adjustments to his home psychiatric medication, quetiapine. By hospital day nine, the patient's mental status improved, and he was discharged safely back to his nursing home.

## Discussion

Fahr’s disease progresses with worsening neurological dysfunction, bilateral basal ganglia calcification, and systemic or mitochondrial conditions (including endocrinopathies) [14]. The affected patients usually present with a family history of autosomal dominant inheritance. The patient described in this case study presented with Fahr’s disease and comorbidities, including schizophrenia and toxic metabolic encephalopathy [4]. He appeared with altered mental status despite showing improvements in clinical symptoms and biochemical outcomes. The patient’s schizophrenia did not require urgent medical management in the absence of active hallucinations and a history of medication adherence. The medical management subsequently aimed to mitigate sepsis progression and Fahr’s disease.

The diagnostic management of encephalopathy from a systemic illness warrants the assessment of its contributing factors (including toxic-metabolic and drug-induced encephalopathies) and their neurological manifestations [15-17]. The brain MRI findings in acute sepsis often indicate ischemic lesions (in the cortex and hippocampus regions), cytotoxic edema, vasogenic edema, posterior reversible encephalopathy syndrome, white matter disruption, and frontal cortical atrophy [18]. Other central nervous system manifestations include dysfunctional neurotransmission, BBB impairment, microgliosis, and systemic insults (i.e. persistent hyperglycemia, severe hypoxemia, and prolonged inflammation). The assessment of clinical deterioration and treatment response, in this case, proved challenging due to undetermined infection severity, neurological compromise, and sepsis-associated encephalopathy. The neurological consultation guided the administration of supportive therapy that resulted in substantial clinical improvement; however, the delayed cognitive response of the patient correlated with pre-existing comorbidities. The contemporary literature reveals the high incidence of long-term cognitive dysfunction and delayed recovery in patients with delirium due to sepsis-associated encephalopathy [19]. In addition, apoptosis, brain inflammation, altered brain signaling, BBB breakdown, endothelial activation, and vascular damage increase the risk and incidence of deep coma and all-cause mortality [20]. Fahr’s disease in this case worsened aspiration pneumonia, physical deconditioning, delirium, oropharyngeal dysphagia, and other potential complications from comorbid conditions [21].

This case study could not determine the hospitalization history of the patient; however, the diagnostic assessment did not rule out the possible impact of SARS-CoV-2 infection on overall functional/cognitive decline and other neurological manifestations. A similar case study by Demir et al. elaborated on Fahr’s syndrome of an elderly patient with SARS-CoV-2 pneumonia who experienced tonic-clonic convulsions in the intensive care unit [22]. The study, however, did not rule out the possible impact of COVID-19-related inflammation and hydroxychloroquine therapy on the reported seizure. These findings warrant the regular monitoring of neurological symptoms in similar patient scenarios for determining their etiology and improving the treatment outcomes. The outcomes of this case study revealed the deleterious impact of oxidative stress, severe inflammation, and immune insult from sepsis in patients with SARS-CoV-2 infection, Fahr’s syndrome, and neurological complications [23-26]. This study proved to be another potential opportunity to explore complex pathological pathways contributing to the clinical presentation of a patient with COVID-19 infection, Fahr’s disease, acute delirium, and aspiration pneumonia.

## Conclusions

The patient in this case study presented with aspiration pneumonia, sepsis, toxic metabolic encephalopathy, acute delirium, and neurocognitive decline potentiated by SARS-CoV-2 infection and Fahr’s disease. The aspiration pneumonia and its clinical complications correlated with the patient’s history of Fahr’s disease and its metabolic manifestations. This case study elaborated on possible mechanisms of sepsis-induced inflammatory changes and their role in triggering neurological complications. The complex pathophysiology of Fahr’s syndrome and toxic metabolic encephalopathy restricted the assessment of infection severity and treatment response. The case outcomes also emphasized the long-term neurological complications of COVID-19 infection in the context of Fahr’s disease. The clinical studies emphasize the adverse impact of SARS-CoV-2 infection on the central nervous system of patients with preexisting neurological complications; however, the underlying pathophysiology is not completely explored. This case report emphasizes the need for monitoring the neurological status of COVID-19-infected patients with preexisting neurological impairments and severe sepsis. Future research should aim to unravel mechanisms and pathways leading to progressive neurocognitive deterioration in patients with systemic inflammation and a known history of neurological disorders.
